# When eating healthy is not healthy: orthorexia nervosa and its measurement with the ORTO-15 in Hungary

**DOI:** 10.1186/1471-244X-14-59

**Published:** 2014-02-28

**Authors:** Márta Varga, Barna Konkolÿ Thege, Szilvia Dukay-Szabó, Ferenc Túry, Eric F van Furth

**Affiliations:** 1 Institute of Behavioral Sciences, Semmelweis University, 1089 Nagyvárad tér 4, Budapest, Hungary; 2 Department of Psychology, University of Calgary, 2500 University Drive NW, Calgary, AB T2N 1N4, Canada; 3 Center for Eating Disorders Ursula, Veursestraatweg 185, 2264 EG Leidschendam, the Netherlands; 4 Department of Psychiatry, Leiden University Medical Center, Albinusdreef 2, 2333 ZA Leiden, the Netherlands

**Keywords:** Eating disorders, Orthorexia nervosa, Assessment, Psychometric properties, Eating habits, Lifestyle habits

## Abstract

**Background:**

For a better differential diagnosis of eating disorders, it is necessary to investigate their subtypes and develop specific assessment tools to measure their specific symptoms. Orthorexia nervosa is an alleged eating disorder in which the person is excessively preoccupied with healthy food. The ORTO-15, designed by Donini and colleagues, is the first and only at least partially validated instrument to measure this construct. The aims of the present study were to examine the psychometric properties of its Hungarian adaptation (ORTO-11-Hu), and to investigate its relationship to food consumption and lifestyle habits in order to contribute to a better description of the phenomenon.

**Methods:**

The ORTO-11-Hu, a lifestyle habits questionnaire, a food choice list indicating foods the participants choose to consume, and ten additional orthorexia-related questions were administered to a group of 810 Hungarian participants (89.4% female) aged between 20 and 70 (M = 32.39 ± 10.37 years).

**Results:**

Confirmatory factor analysis suggested a single factor structure for the 11-item shortened version of the instrument. Internal consistency of the measure was adequate (Cronbach’s alpha = 0.82). No significant differences were found between males and females on the ORTO-11-Hu. Age and body mass index were significantly associated with a tendency towards orthorexia nervosa. Additional orthorexia-related features were significantly correlated with ORTO-11-Hu scores: orthorexia nervosa tendency was associated not only with healthier food choices (eating more whole wheat cereals, less white wheat cereals, more fruit and vegetables) but with shopping in health food stores, as well as with some healthy lifestyle habits (more sports activity, specific dietary behaviors, and less alcohol intake). Individuals with higher orthorexia nervosa tendency also reported a greater tendency to advocate their healthy diet to their friends and family members.

**Conclusions:**

These results provide evidence for the reliability of ORTO-11-Hu and some support for the construct validity of the instrument. The present study also contributes to the establishment of (diagnostic) criteria for this new subtype of eating disorders.

## Background

To achieve a better differential diagnosis of eating disorders, investigating the various subtypes and developing specific assessment tools to measure specific symptoms is necessary. Orthorexia nervosa (ON) is an alleged eating disorder in which the person is excessively preoccupied with healthy food. First described by Bratman in 1997, ON entails a fixation on healthy food or a health food dependence [1]. The term orthorexia nervosa arises from the words orthos (=accurate) and orexis (=hunger) meaning obsession with healthy food and proper nutrition. Fears and worries about health, eating, and the quality of food are significant.

In extreme cases, the obsessive and compulsive characteristics of ON become pathological and dominate a person’s life. The preoccupation with quality of food and eating healthy comprise the principal elements of this disorder. The pathological obsession with biologically pure food and shops which sell it leads to a special lifestyle. Stringent dietary restrictions and eating plans, combined with a personality and attitude of superiority and obsessive-phobic behavioral characteristics define the core of ON. Transgressing the dietary rules leads to intense anxiety, feelings of guilt and shame and is followed by even more stringent dietary restrictions [1,2].

At present, ON is not a formal disorder, but more and more clinical reports related to it have appeared as it becomes more and more familiar to health care professionals [3]. There is no consensus on the categorization of ON among mental disorders or even if it is a mental disorder. ON was debated on whether or not it should have a place in the recently published DSM-5 [4]. In sum, ON is a new and controversial concept.

Two instruments have been developed to assess ON. The Bratman test is based mostly on clinical experience, and its validity has not been investigated by the author himself or by others. In 2005, Donini and colleagues developed the ORTO-15 based on Bratman’s test [5]. However, this instrument also includes items not specifically characteristic of ON making the face validity of the test doubtful. The ORTO-11 scale, the only foreign language (Turkish) adaptation of the ORTO-15 to date, was designed after omitting four non-ON-specific items based on statistical considerations [6]. In addition, the psychometric properties of the ORTO-15 have only been partially examined by the original test authors. Due to not having a well-defined group of individuals with ON for this purpose, Donini and colleagues proposed a cutoff point to distinguish between individuals with or without ON based on the interrelationship of ORTO-15 scores with obsessive-phobic personality traits as measured by the Minnesota Multiphasic Personality Inventory. A further limitation of the documentation is the inadequate description of the scoring method.

Some clinical studies have also been carried out to better understand the characteristics of ON [7,8]. These suggest that the difference between an ON sufferer and a health-conscious person include the extreme preoccupation with and the judging attitude towards others who do not follow a healthy diet. These studies also provide recommendations for distinguishing ON from anorexia nervosa, psychosis, and poisoning fears.

The aim of the present study was to describe the adaptation process of the ORTO-15 into Hungarian and to investigate the psychometric properties of this version in a large Hungarian sample. We investigated the relationship between the ORTO-15 and ON features such as food choices and nutritional supplement use, alcohol, drug, and cigarette use, and some demographic variables, and added supplemental ON specific questions to examine external criteria for ON. We hypothesized that individuals who have a tendency for ON, in addition to making healthier food choices, refuse to use drugs, alcohol, cigarettes, and take more nutritional supplements, engage in more sports activity and have more dietary restrictions than those without a tendency for ON. Beyond the psychometric aspects, we also intended to improve the knowledge in the field concerning cultural differences, the investigating of which is largely missing from the literature on ON.

## Methods

### Sample and procedure

The study protocol was approved by the Ethics Committee of Semmelweis University (registration number 4498–0/2011-EKU; 410/PI/11). Participants were recruited from different universities in Hungary: students from Semmelweis University, Eötvös Loránd University, the University of Pécs, and the University of Debrecen. The data were collected via internet using the online test battery developed by the authors. Participants received the link to the research called ‘Nutrition and health’ in an email from the secretary at each university. In total, 810 individuals participated in the study (724 women, 86 men; 89.4% vs. 10.6%). The mean age of the respondents was 32.39 ± 10.37 years (31.48 ± 11.68 for males and 30.38 ± 10.20 for females). Body-mass index of the participants ranged between 14.88 and 56.06, with a mean of 23.20 ± 4.89.

The majority of participants comprised students from the following areas: dietetics (N = 69, 8.5%), medicine (N = 107, 13.2%), other health-related professions (nursing, physiotherapy, midwifery, optometry, physical education, and sport sciences; N = 240, 29.6%), and from other fields (sociology, social work, teaching; N = 33, 4.1%). Other participants included graduated professionals: dieticians (N = 26, 3.2%), medical doctors (N = 18, 2.2%), other healthcare professionals (N = 55; 6.8%), and non-healthcare professionals (N = 262, 32.3%).

All instruments were administered online in order to reach as many participants as possible. Participants completed an informed consent form, entered demographic information, filled in the Hungarian translation of the ORTO-15 and a checklist of food choices, and several ON-specific questions developed for the purpose of this study – based on the clinical experiences and observations from other authors [1,2,7]. Finally, participants were asked about their lifestyle (sports activity, dieting) and other foodstuff consumption habits (nutritional supplements, medication, alcohol, drugs, and smoking).

### Instruments

#### ***Orthorexia nervosa***

ORTO-15 is a measure instrument for ON comprising 15 multiple-choice items [5]. This instrument is based on Bratman’s orthorexia model combined with MMPI obsessive-phobic personality trait questions [1,9]. ORTO-15 is a self-report questionnaire with a 4-point Likert-scale (always, often, sometimes, never). The items address the selection, preparation, consumption, and effect of, and attitude towards presumed to be healthy food. The original test authors assumed three underlying factors: the cognitive-rational (items 1,5,6,11,12,14), the clinical (items 3,7,8,9,15) and the emotional (items 2,4,10,13) components of ON [5]. The authors also tested the efficacy, sensitivity, specificity, positive and negative predictive value of three different threshold values for ORTO-15: <35, <40, and <45. Compared to the other two cutoff points, 40 (lower scores refer to more ON features) was considered to be the most appropriate for distinguishing between individuals with and without ON. In one sample, this cutoff had 73.8% efficacy, 55.6% sensitivity, 75.8% specificity, 20.5% positive predictive value, 93.8% negative predictive value; while in another sample it showed 75% efficacy, 100% sensitivity, 73.6% specificity, 17.6% positive predictive value and 100% negative predictive value [5].

For developing the Hungarian version of the ORTO-15, the first and the third author of the present study translated ORTO-15 into Hungarian. No new expressions were added nor were changes made to the original structure. The final version was created by using the expert opinions of two clinical psychologists. A back translation was conducted by an independent bilingual translator, and was compared to the original test by yet another professional; no significant changes were necessary. The final version of the scale was administered to 20 people to evaluate the clarity of the items.

#### ***Lifestyle habits***

Participants were asked about their sports activity, dietary restrictions, caffeine, alcohol, drug, cigarette, medication, and nutritional supplement use. Frequency of sports activity was assigned to the following categories: do not engage in any sport, casually, regularly as a hobby, compete regularly. Smoking status was coded as current, former, and nonsmoker. Alcohol, caffeine, medication, and nutritional supplement use were addressed with yes/no type questions.

#### ***Food choice list***

Individuals were asked about the type of foods they usually eat. Food categories were developed by a dietician based on the nutrition pyramid [10]. Participants rated the frequency of their consumption of given food types. The food choice list contained the following food categories: whole wheat bread, white bread (rated on a 3-point scale: daily, weekly, monthly or rarely), and vegetables plus fruits (rated on a 6-point Likert-scale: several times a day, once daily, several times per week, once a week, once a month, never, or very rarely). Individuals were also asked whether and how often they shop at stores which sell primarily healthy food (regularly, occasionally, never).

#### ***Supplemental orthorexia nervosa-related questions***

Ten further questions were added to the ORTO-15 based on the descriptions of reviews and clinical reports about ON [1,2,7]. These were related to food consumption habits (so-called health food, timing, food color choices), prejudices and attitudes related to obesity and problems with controlling desires. Individuals had to rate whether they agree or not with the items.

Supplemental orthorexia nervosa-related questions used in this study:

1. I consume only healthy foods.

2. I always eat according to my eating schedule.

3. Sexuality plays an important role in my life.

4. Being overweight is a sign of weakness.

5. I avoid food with specific colors.

6. I disapprove of people who cannot overcome their desires.

7. I think most people can be blamed for their own diseases.

8. I always eat the same meals.

9. I am critical of people who don’t follow the rules of a healthy lifestyle.

10. I spend a large amount of time preparing my meals.

### Statistical analyses

In order to evaluate the factor structure of the Hungarian version of the ORTO-15, four confirmatory factor analytic models were tested. Model 1 was a three-factor (‘cognitive-rational attributes’, ‘clinical attributes’, and ‘emotional attributes’) model based on the assumptions of the original authors of the instrument [5]. Model 2 was a single-factor model with one factor responsible for all 15 item responses. Since neither of the two models adequately fit the data, a shortened version of the instrument was developed by omitting four items with the lowest item-total correlations and factor loadings. The factor structure of this 11-item version was evaluated by a third confirmatory analytic model using a single-factor solution. Finally, a fourth model was also evaluated containing covariances suggested by modification indices from Model 3.

Internal reliability of the original and the shortened versions was evaluated by calculating Cronbach’s alpha coefficients. Normality of the continuous variables was tested by the Shapiro-Wilk W statistics. Because of the non-normal distribution of our continuous variables, relationships among them were analyzed using nonparametric methods. The Mann–Whitney and the Kruskal-Wallis tests were applied to evaluate the relationships between categorical and continuous variables, while Spearman correlation coefficients were calculated to estimate the strength of the associations between two continuous variables. Confirmatory factor analyses were conducted using AMOS version 20.0, while all other statistical procedures were carried out using SPSS 20.0 software.

## Results

### Factor structure, item analysis, and reliability

**Figure 1:**
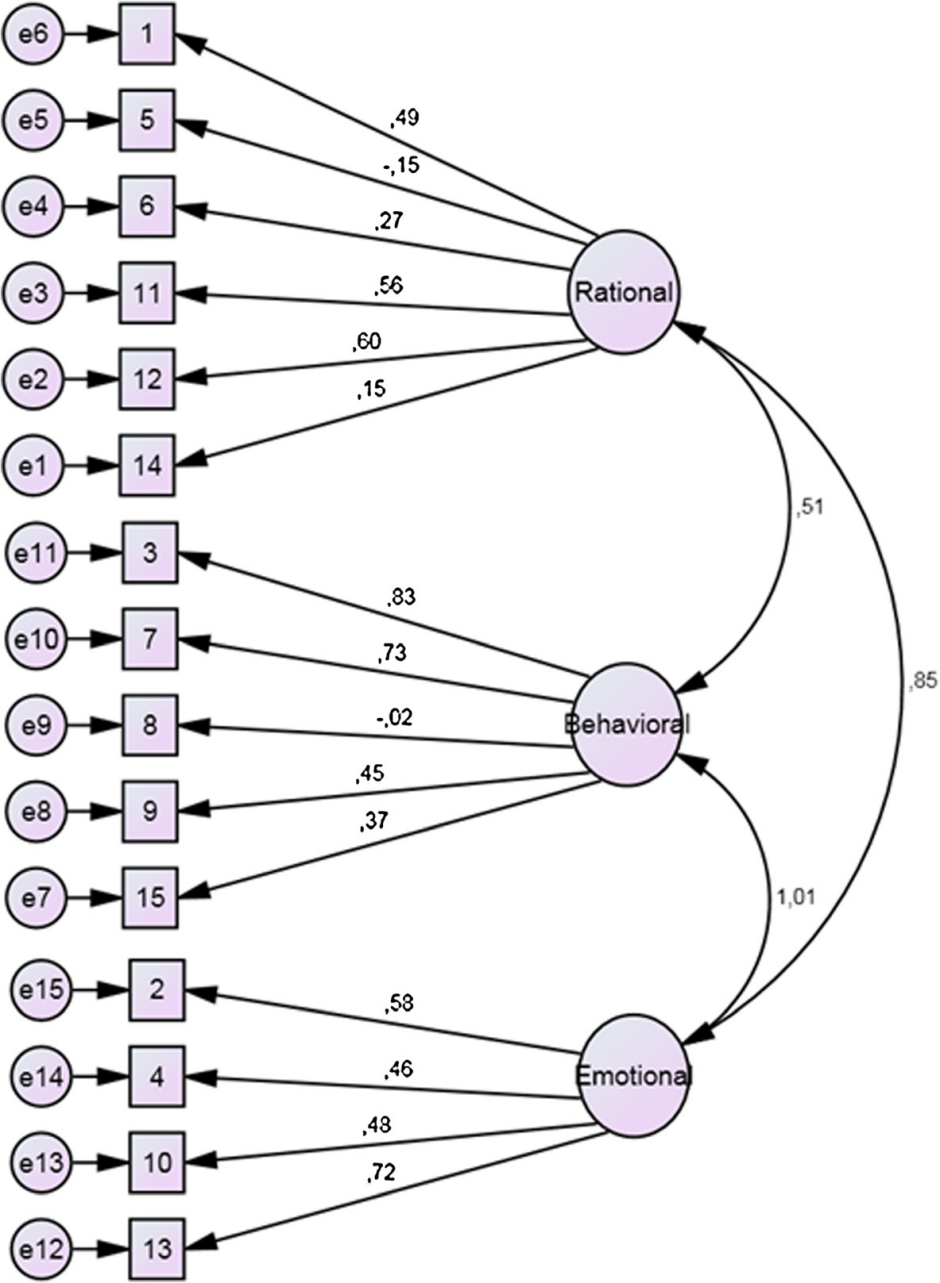
**The factor structure of ORTO-15 as hypothesized by Donini and colleagues (displayed values are unstandardized regression weights from the present sample).** Legend: Big circles represent factors, squares represent items, while small circles represent error terms.

First, we tested whether our data fit well with the factor structure hypothesized by the original authors of the instrument (Figure 1). The confirmatory factor analytic investigation revealed that the three-factor solution (Model 1), suggested by Donini and colleagues, had to be rejected (χ^2^ = 931.2; p < 0.001; CMIN/DF = 10.7; CFI = 0.72; TLI = 0.66; RMSEA = 0.11; PCLOSE < 0.001). The single-factor solution (Model 2) showed slightly lower goodness of fit indices (χ^2^ = 1080.8; p < 0.001; CMIN/DF = 12.0; CFI = 0.67; TLI = 0.62; RMSEA = 0.12; PCLOSE < 0.001); therefore, this model was rejected as well.

**Figure 2:**
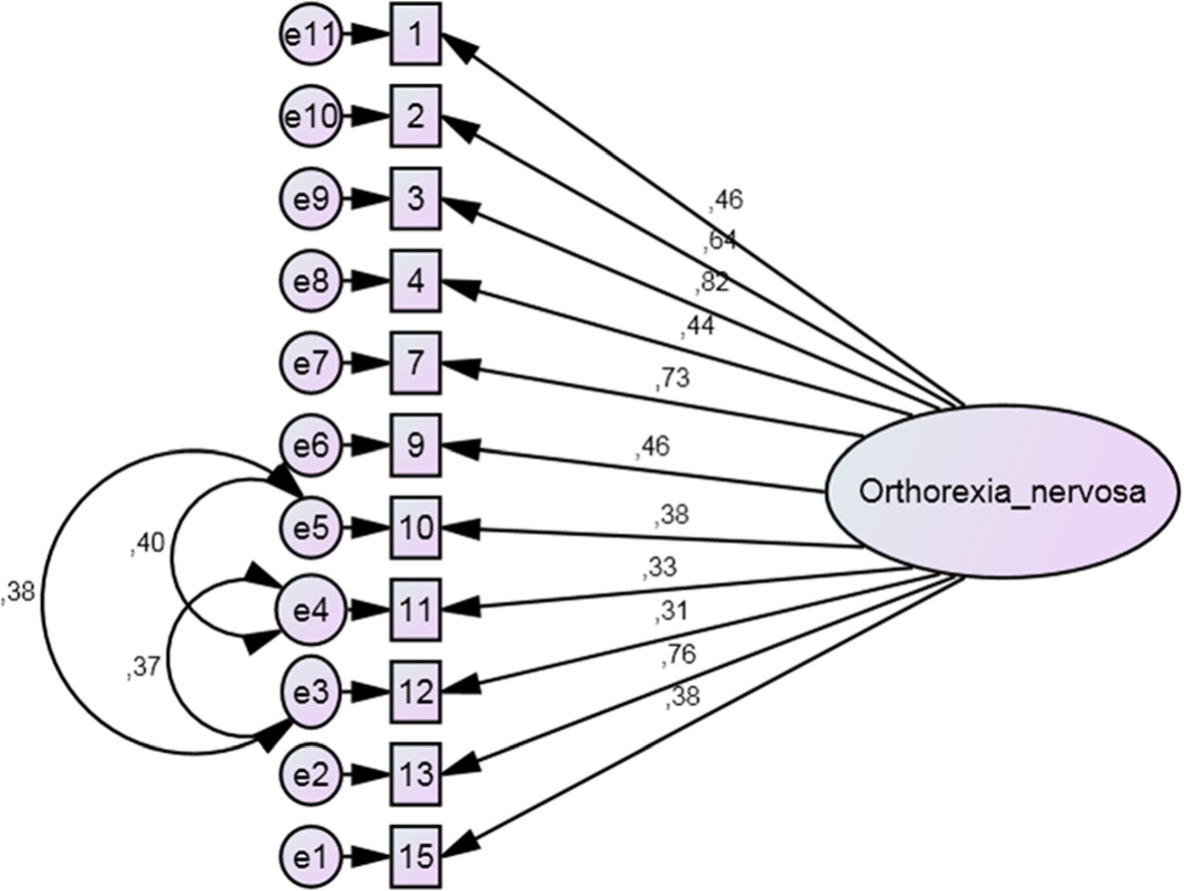
**The best fitting, final model of the shortened, 11-item Hungarian version of ORTO-15 (displayed values are standardized regression weights).** Legend: Big circles represent factors, squares represent items, while small circles represent error terms.

Because of the poor fit indices of the first two models, an item analysis was conducted to evaluate the appropriateness of each item (Table 1). Based on the particularly low item-total correlations and factor loadings from the second confirmatory factor analytic model, we decided to omit item numbers 5, 6, 8, and 14. A further confirmatory factor analysis was conducted to examine the goodness of fit of the shortened version’s single-factor structure. Although this solution showed better fit indices (χ^2^ = 530.8; p < 0.001; CMIN/DF = 12.1; CFI = 0.81; TLI = 0.76; RMSEA = 0.12; PCLOSE < 0.001) compared to the previous two models, the indicators still remained unsatisfactory. Therefore the variances of the error terms were analyzed through modification indices. Following the cutoff criteria of modification indices equal to or higher than 50, three covariances between error terms were incorporated into a fourth model generating a new single-factor structure (Figure 2). The error terms that were correlated were items (modification indices between parentheses) 10/11 (116.5), 10/12 (107.0), and 11/12 (96.4). The fit indices of this fourth model proved to be acceptable (χ^2^ = 230.8; p < 0.001; CMIN/DF = 5.63; CFI = 0.92; TLI = 0.90; RMSEA = 0.076; PCLOSE < 0.001). Internal consistency of both the original 15-item and the shortened 11-item version proved to be adequate (Cronbach’s alpha = 0.78 and 0.82, respectively). The full text of the original ORTO-15 and its Hungarian version, ORTO-11-Hu can be found in the Table 2.

**Table 1:** Results of the item analysis of ORTO-15 in our sample

	**Mean**	**Standard deviation**	**Original, 15-item version**			**Final, 11-item version**		
			**Corrected item-total correlation**	**Cronbach’s alpha if item deleted**	**Standardized factor loadings in Model 2**	**Corrected item-total correlation**	**Cronbach’s alpha if item deleted**	**Standardized factor loadings in Model 4**
Item 1	2.09	0.94	0.47	0.76	0.48	0.43	0.81	0.46
Item 2	1.29	0.63	0.46	0.77	0.63	0.53	0.80	0.64
Item 3	1.76	0.94	0.57	0.75	0.80	0.65	0.79	0.82
Item 4	2.08	0.88	0.49	0.76	0.46	0.44	0.81	0.44
Item 5 (reversed)	2.71	0.81	0.11	0.79	0.04	–	–	–
Item 6	2.81	0.73	0.22	0.78	0.08	–	–	–
Item 7	1.42	0.82	0.51	0.76	0.71	0.58	0.80	0.73
Item 8 (reversed)	2.33	0.66	0.18	0.78	-0.07	–	–	–
Item 9	2.73	0.86	0.32	0.77	0.46	0.40	0.81	0.46
Item 10	2.24	0.92	0.52	0.76	0.43	0.48	0.81	0.38
Item 11	2.31	0.94	0.45	0.76	0.39	0.43	0.81	0.33
Item 12	2.96	0.83	0.45	0.76	0.37	0.42	0.81	0.31
Item 13	2.06	0.97	0.60	0.75	0.77	0.66	0.79	0.76
Item 14	3.11	0.82	0.10	0.79	0.07	–	–	–
Item 15	2.32	0.62	0.30	0.78	0.37	0.33	0.82	0.38

**Table 2:** Full text and scoring grid of the original ORTO-15 and the Hungarian version, ORTO-11-Hu

**English original**	**Hungarian version**
1. When eating, do you pay attention to the calories of the food?	1. Ha eszik, figyel az étel kalóriatartalmára?
2. When you go in a food shop do you feel confused?	2. Ha bemegy egy élelmiszerboltba, zavartnak érzik magát?
3. In the last 3 months, did the thought of food worry you?	3. Az utóbbi három hónapban az étel gondolata aggasztotta Önt?
4. Are your eating choices conditioned by your worry about your health status?	4. Ételválasztásának feltételévé vált-e az egészségi állapota miatti aggódás?
5. Is the taste of food more important than the quality when you evaluate food?	*5. Az étel íze fontosabb, mint a minősége, amikor az ételt értékeli?*
6. Are you willing to spend more money to have healthier food?	*6. Hajlandó több pénzt költeni egészségesebb ételekre*?
7. Does the thought about food worry you for more than three hours a day?	7. Az ételekről szóló gondolatok naponta több mint három órán át aggasztják?
8. Do you allow yourself any eating transgressions?	*8. Megengedi magának az étkezési szabályok áthágását?*
9. Do you think your mood affects your eating behavior?	9. Gondolja, hogy hangulata befolyásolja étkezését?
10. Do you think that the conviction to eat only healthy food increases self-esteem?	10. Gondolja, hogy az a meggyőződés, hogy csak egészséges ételt egyen, növeli az önbecsülést?
11. Do you think that eating healthy food changes your life-style (frequency of eating out, friends, …)?	11. Gondolja, hogy az egészséges étel evése megváltoztatja az életstílusát? (az otthon kívül evés gyakorisága, barátok…)?
12. Do you think that consuming healthy food may improve your appearance?	12. Gondolja, hogy egészséges ételek fogyasztása javíthatja a megjelenését?
13. Do you feel guilty when transgressing?	13. Bűnösnek érzi magát, ha kihágást követ el?
14. Do you think that on the market there is also unhealthy food?	*14. Gondolja, hogy a piacon vannak egészségtelen ételek is?*
15. At present, are you alone when having meals?	15. Általában egyedül van, amikor eszik?
Answer categories:	Válaszlehetőségek:
Always – Often – Sometimes – Never	Mindig - Gyakran – Néha - Soha
**Scoring grid for ORTO-15 test responses, items responses**	
Always – Often — Sometimes — Never	
Items	Scooring
2-5-8-9	4 3 2 1
3-4-6-7-10-11-12-14-15	1 2 3 4
1-13	2 4 3 1

### Descriptive statistics and relationships with sociodemographic variables

The mean score on the ORTO-11-Hu was 28.83 with a standard deviation of 3.15, while that of the original 15-item version was 37.87 ± 3.93. Using the original cut-off point of 40 on the original 15-item version, 74.2% (N = 601) of this study’s sample had a tendency for ON. Further, no significant sex differences were found on the ORTO-11 (females vs. males: 28.81 ± 3.14 vs. N. 29.02 ± 3.25). The relationship of ORTO-11-Hu scores with age and body mass index was also examined. There was a statistically significant but weak correlation present between orthorexia scores and age (r = 0.13, p < 0.01) but no significant association was found concerning body mass index.

### Relationships to other ON-related constructs as support for construct validity

#### ***Health behaviors***

Participants engaging in more sports activity had lower ORTO-11-Hu scores (meaning a higher tendency for ON) compared to people who did not engage in any sport. There were no significant differences in ORTO-11-Hu scores with respect to smoking status, caffeine use, and medicine and nutrition supplement intake. However, participants who never drank alcohol had a higher tendency for ON (lower scores) compared to others consuming alcohol. Individuals on special diets also showed significantly lower scores on ORTO-11-Hu (stronger ON tendency) than non-dieting individuals. Finally, individuals with a greater tendency to encourage relatives and friends to follow their supposedly healthy diet reported a higher tendency for ON compared to individuals who did not try to convince others (see Table 3 for details).

**Table 3:** Relationship of ORTO-11-Hu scores with health behaviors

**Health behavior**	**Frequency**	**ORTO-11-Hu scores (M ± SD)**	**Statistics**	**Significance**
Sports	Regularly/Competitive	29.00 ± 2.73	χ^2^ = 17.08	0.001
	Regularly as hobby	28.27 ± 3.28		
	Occasionally	28.97 ± 3.02		
	Not at all	29.37 ± 3.11		
Smoking	Current	29.05 ± 3.28	χ^2^ = 1.93	0.380
	Former	28.90 ± 3.01		
	Never	28.72 ± 3.15		
Caffeine intake	Never	28.34 ± 3.34	χ^2^ = 9.88	0.079
	Rarely	28.56 ± 3.03		
	Monthly	28.36 ± 2.81		
	Weekly	28.83 ± 2.98		
	Several times per week	28.77 ± 2.98		
	Daily	29.14 ± 3.16		
Medication use	Yes	29.02 ± 3.52	Z = -1.68	0.094
	No	28.77 ± 3.03		
Nutrition supplement use	Yes	28.67 ± 3.20	Z = -1.31	0.191
	No	28.95 ± 3.15		
Alcohol use	Never	28.10 ± 3.68	χ^2^ = 13.02	<0.05
	Rarely	28.49 ± 2.97		
	Monthly	29.18 ± 3.01		
	Weekly	29.14 ± 3.18		
	Several times per week	29.47 ± 3.60		
	Daily	28.80 ± 1.30		
Special diet	Yes	27.86 ± 3.38	Z = -4.18	<0.001
	No	29.07 ± 3.04		
Convincing others to follow diet	Yes	28.55 ± 3.13	Z = -3.04	<0.01
	No	29.19 ± 3.13		

#### ***Food choices***

Individuals eating whole wheat cereals were more frequently characterized by higher ON tendency compared to others eating whole wheat cereals less often. In addition, participants who more frequently ate white wheat cereals scored higher on ORTO-11-Hu than others who ate white wheat cereals occasionally. Concerning vegetable and fruit consumption, only tendencies were observed in the expected directions: ORTO-11-Hu scores were lower (higher ON tendency) for individuals eating fruits and vegetables. Finally, ORTO-11-Hu scores were significantly lower in the groups of individuals who more often visited shops selling healthy food compared to those who did not shop at such stores (see Table 4 for details).

**Table 4:** Relationship of ORTO-11-Hu scores with food choices

**Food type**	**Frequency of consumption**	**ORTO-11-Hu scores (M ± SD)**	**Statistics**	**Significance**
Whole wheat cereals	Rarely or monthly	28.94 ± 3.19	χ^2^ = 8.21	<0.01
	Weekly	29.12 ± 2.81		
	Daily	28.28 ± 3.45		
White wheat cereals	Rarely or monthly	27.63 ± 3.26	χ^2^ = 17.41	<0.001
	Weekly	28.75 ± 3.13		
	Daily	29.09 ± 3.08		
Vegetables	Rarely or never	31.22 ± 1.98	χ^2^ = 9.41	<0.1
	Monthly	28.77 ± 3.35		
	Weekly	29.21 ± 2.95		
	Several times per week	28.95 ± 3.08		
	Daily	28.78 ± 3.08		
	Several times per day	28.46 ± 3.35		
Fruits	Rarely or never	29.35 ± 3.79	χ^2^ = 9.49	<0.1
	Monthly	28.97 ± 3.06		
	Weekly	29.35 ± 2.88		
	Several times per week	29.07 ± 2.81		
	Daily	28.87 ± 3.28		
	Several times per day	28.29 ± 3.31		
Visiting health food stores	Never	29.10 ± 2.95	χ^2^ = 9.33	<0.01
	Occasionally	28.56 ± 3.40		
	Regularly	27.86 ± 3.13		

#### ***Supplemental orthorexia nervosa-related questions***

The data confirmed the link between a tendency towards ON and scores on the supplemental questions based on the clinical literature on this phenomenon. ORTO-11-Hu scores were significantly associated with the consumption of healthy food and eating according to a fixed schedule, indicating that respondents consuming mainly healthy food and following a strict eating schedule scored lower on ORTO-11-Hu (meaning a higher tendency for ON). Individuals who scored lower on ORTO-11-Hu considered being overweight more often as a sign of weakness, and were more prone to avoid foods with specific colors and to blame people for their diseases. In addition, individuals with a higher tendency for ON more often judged people negatively who could not overcome their desires and who did not follow the rules of healthy nutrition. Finally, respondents with more ON features had a higher tendency to eat the same food everyday and to spend a large amount of time with preparation of meals (see Table 5 for details).

**Table 5:** Relationship of ORTO-11-Hu scores with the supplemental orthorexia nervosa-related questions

	**ORTO-11-Hu scores (M ± SD)**		**Statistics (Z)**	**Significance**
	**Agree**	**Disagree**		
Consumption of only health food	27.20 ± 3.14	28.97 ± 3.11	-4.19	<0.001
Eating according to a fixed eating schedule	28.09 ± 3.28	29.08 ± 3.06	-3.70	<0.001
Thinking of overweight as a sign of weakness	28.08 ± 3.53	29.39 ± 2.70	-5.27	<0.001
Avoiding food with specific colors	27.69 ± 3.21	28.95 ± 3.12	-3.24	<0.001
Judging people negatively who cannot overcome their desires	28.26 ± 3.41	29.07 ± 3.00	-2.58	<0.010
Blaming people for their diseases	27.97 ± 3.31	29.25 ± 2.98	-5.43	<0.001
Eating the same food everyday	27.88 ± 3.58	29.14 ± 2.93	-4.18	<0.001
Judging people who do not follow the rules of healthy nutrition	27.60 ± 3.75	29.08 ± 2.95	-4.09	<0.001
Spending a large amount of time with preparation of meals	26.84 ± 3.50	29.22 ± 2.92	-7.14	<0.001

## Discussion

Orthorexia nervosa is a recently proposed eating disorder where the quality of food becomes an obsession. Until now, only some basic steps have been made to operationalize this construct: the psychometric status of the first and only measurement instrument (ORTO-15) for ON is still unclear. The aim of this study was to contribute to the scientific literature on the assessment of this possible new eating disorder and to describe its clinical features.

The confirmatory factor analytic findings of this study did not provide support for the three-factor structure recommended by the original test authors [5]. Our final analysis suggested a one-factor solution with 11 items (item numbers 1, 2, 3, 4, 7, 9, 10, 11, 12, 13, and 15) omitting item numbers 5, 6, 8, and 14 from the original ORTO-15 in this Hungarian sample (thus creating ORTO-11-Hu). Internal consistency of this shortened 11-item version proved to be adequate.

To provide our results with international context, it is worth mentioning that the Turkish adaptation of ORTO-15 also led to an 11 item-questionnaire with a one-factor solution. However, the item-composition of the Turkish version is somewhat different: it contains items 3, 4, 5, 6, 7, 8, 10, 11, 12, 13, 14 from ORTO-15. The internal consistency of the Turkish ORTO-11 (0.62) [6] was lower than that of the Hungarian version in the present sample. Concerning item composition, the items that were dropped from the Turkish and the Hungarian questionnaire are different. The four items excluded from the Hungarian one were related to quality preference (item 5) and the financial aspect of food choices (item 6), the transgression of dietary rules (item 8) and beliefs about unhealthy food at the market (item 14). The Turkish version omitted the following items: the calorie aspect of food (item 1), finding food shopping confusing (item 2), mood specificity (item 9), and a preference for eating alone (item 15).

Cultural differences may explain at least some of these differences, including the fact that Turkish society is characterized by collectivism, contrary to Hungary which has a strongly individualist culture. Hungarians tend to view themselves mainly as individuals emphasizing the needs of a single person. In Turkey, however, people tend to view themselves mainly as members of groups (families, work units, tribes, nations), and usually consider the needs of the group to be more important than the needs of individuals [11]. Another important difference may be related to the Muslim religion that has different values compared to Christianity (restrictions in the consumption of certain food types, the meaning of fasting, attitudes towards hedonism, habits of eating in the family, etc.). These differences might also be associated with general cross-cultural differences in the expression of eating disorders, and with the availability and quality of different foods. Symptoms of ON may thus vary across different countries and cultures, which underlines the importance of further cross-cultural research on this phenomenon.

Our final confirmatory factor analytic model contained three covariances between error terms suggested by modification indices from previous analyses. The necessity of the inclusion of these associations might be explained by the fact that item numbers 10, 11, and 12 contain more words than the other items in the Hungarian version of ORTO-15. These items are less understandable because of their multiple complex structures (a characteristic of the Hungarian language). The beginning of these three sentences is also the same, and the end of the sentences contains the main message of the item. Further, all three sentences contain the expression ‘eating healthy food’. A further relevant factor can be the difficulty of distinguishing between self-esteem, lifestyle, and appearance in the context of eating healthy food.

Concerning the cutoff score, highly unexpected results were found in the present sample. Setting the cutoff score at 40 for defining the occurrence of ON, as suggested by the original test authors [5], yielded a 74.2% point prevalence in our sample (lower scores refer to higher tendency for ON). This high prevalence rate is similar to the prevalence rates found in other studies employing the same cutoff point [12-15]. The reason for such a high percentage can be either that the cutoff point is not correct or the validity of the scale is poor. The finding that scores on the ORTO-11-Hu were significantly associated with ON-related behaviors in our sample supports the previous opinion of the inadequacy of the cutoff point in addition to the construct validity of the instrument. Further research should contribute to the establishment of a reliable cutoff point to distinguish between individuals with and without ON.

The present findings also showed that the association between ORTO-11-Hu scores, age, and body mass index was statistically significant but negligible. These results are in line with the diverse results of other studies which reported no differences in age [6] or body mass index [6,13,16,17]. In other studies, lower age was found to be associated with higher scores on ORTO-15 [12,14]. However, these studies were conducted among medical students, so the level of education may be a moderator in this relationship: several studies report that an earlier stage of university studies is associated with orthorectic features but this relationship weakens towards the end of studies [18]. Only the study of Donini and colleagues [5] reported data inconsistent with these results. Consistent with our present findings, most of the former studies also did not find a significant association between ON features and body mass index [6,13,16,17]. As some authors suggest, body mass index can predict orthorectic behaviors in combination with other variables such as medical reasons, dieting, and healthy nutrition [6]. However, a study reported higher body mass index scores associated with lower ORTO-15 scores among medical students [14], meaning that higher body mass index is related to orthorectic behaviors in this special population.

In the present Hungarian sample, there were no significant differences between male and female individuals, in contrast to the other studies where females had a higher tendency for ON [5,6,14,19]. Because males were underrepresented in our sample, these results provide less reliable data to assess gender-related differences. Further studies should clarify whether our results can be explained by the low rate of male respondents or they reflect some true cultural specialty.

In line with our expectations, individuals in our sample with stronger ON tendency reported eating more whole wheat cereals and less white wheat cereals and more frequent shopping in healthy food stores. The orthorectic features were also related to lifestyle habits such as regular sports activity, more dietary restrictions and less alcohol intake and an inclination to persuade others about the importance of a healthy diet. These results are similar to the descriptions of Bratman [1] regarding ON which includes not only eating habits but other associated lifestyle characteristics.

At present, no comprehensive and standardized criteria for ON exists. Our knowledge in this regard is limited to the description of Bratman, some clinical observations by others, and a few explorative studies conducted with convenience samples from a small number of countries (for a detailed review, see [20]). Since there are no specific external criteria or other validated assessment tools for ON, evaluating the validity of the present instrument is impossible in a strict sense. However, it seemed to be reasonable to use some supplemental questions based on the clinical literature to perform a preliminary validation of the investigated measurement instrument. In line with our expectations, these ON features were significantly associated with ORTO-11-Hu scores supporting some evidence for the validity of the scale. Individuals with higher frequency of eating health food, having a schedule for eating, eating food with a particular color, eating mostly the same type of food and spending lots of time preparing meals showed a stronger tendency for ON. Further, individuals who found sexuality not to be important, considered being overweight as a sign of weakness, and disapproved of people who could not overcome their desires also had a higher tendency for ON. This was also the case for those who believed that people should be blamed for their own diseases, and those who criticized people who did not follow the rules of a healthy lifestyle. These results provide some further support for the construct validity of the ORTO-11-Hu.

Our study has several strengths and limitations. Internet-based sampling may have inherent selection biases towards a younger and more educated sample. Further, the connotation of the name of the study ‘Nutrition and health’ may have attracted individuals for whom nutrition and healthy eating are more important in their everyday life than for individuals in the general population. In addition, all results were based on self-reports not enabling confirmation of the accuracy of responses, which could be especially distorted concerning health behaviors or food choices. However, to the best of our knowledge, this is the first study in the literature investigating the factor structure of an ON scale using confirmatory factor analytic techniques. A further strength of the study is the relatively large sample consisting of individuals with diverse characteristics across occupation and age. In addition, this is the first study to investigate correlates of ON in a diverse and large sample from Eastern Europe.

## Conclusions

We can conclude that the Hungarian version of the ORTO-15 questionnaire has adequate psychometric characteristics, thus providing preliminary support for the use of this instrument. Further research from other countries is needed to improve our knowledge on the psychometric characteristics of the instrument and to better understand the nature and correlates of ON. Future research in this area should identify the place for ON among mental disorders, presumably on the spectrum of eating and obsessive compulsive disorders.

## Competing interests

The authors declare that they have no competing interests.

## Authors’ contributions

VM, FT and EFF designed the study. VM managed the literature searches. Adaptation of the scale was conducted by VM and SZDSZ. VM and BKT conducted the statistical analyses. VM and BKT drafted the manuscript. All authors read and approved the final manuscript.

## Pre-publication history

The pre-publication history for this paper can be accessed here:

http://www.biomedcentral.com/1471-244X/14/59/prepub

## References

[B1] Bratman S The health food eating disorder [http://www.orthorexia.com/original-orthorexia-essay]

[B2] Bratman S Knight D Health food junkies: overcoming the obsession with healthful eating 2000 New York: Broadway Books

[B3] Vandereycken W Media hype, diagnostic fad or genuine disorder? Professionals’ opinions about night eating syndrome, orthorexia, muscle dysmorphia, and emetophobia Eat Disord 2011 19 145 155 10.1080/10640266.2011.551634 21360365

[B4] Fairburn CH Cooper Z Eating disorders, DSM-5 and clinical reality Br J Psych 2011 198 8 10 10.1192/bjp.bp.110.083881 PMC3014461 21200070

[B5] Donini LM Marsili D Graziani MP Imbriale M Cannella C Orthorexia nervosa: validation of a diagnosis questionnaire Eat Weight Disord 2005 10 28 32 10.1007/BF03327537 16682853

[B6] Arusoğlu G Kabakçi E Köksal G Merdol TK [**Orthorexia nervosa and adaptation of ORTO-11 into Turkish**] Turk Psikiyatri Derg 2008 19 283 291 18791881

[B7] Cartwright MM Eating disorder emergencies: understanding the medical complexities of the hospitalized eating disordered patient Crit Care Nurs Clin North Am 2004 16 515 530 10.1016/j.ccell.2004.07.002 15571940

[B8] Zamora CML Bote Bonaechea B García Sánchez F Ríos Rial B [**Orthorexia nervosa. A new eating behavior disorder?**] Actas Esp Psiquiatr 2005 1 66 68 15704033

[B9] Butcher JN Dahlstrom WG Graham JR Tellegen A Kaemmer B The Minnesota multiphasic personality inventory-2 (MMPI-2): manual for administration and scoring 1989 Minneapolis, MN: University of Minnesota Press

[B10] Skerrett PJ Willett WC Essentials of healthy eating: a guide J Midwifery Womens Health 2010 55 6 492 501 10.1016/j.jmwh.2010.06.019 20974411 PMC3471136

[B11] Hofstede G Culture’s consequences: comparing values, behaviors, institutions and organizations across nations 2001 Thousand Oaks, CA: Sage

[B12] Bağci Bosi AT Camur D Güler C Prevalence of orthorexia nervosa in resident medical doctors in the faculty of medicine (Ankara, Turkey) Appetite 2007 49 661 666 10.1016/j.appet.2007.04.007 17586085

[B13] Aksoydan E Comci N Prevalence of orthorexia nervosa among Turkish performance artists Eat Weight Disord 2009 14 1 33 37 10.1007/BF03327792 19367138

[B14] Fidan T Ertekin V Işikay S Kirpinar I Prevalence of orthorexia among medical students in Erzurum, Turkey Compr Psych 2010 51 49 54 10.1016/j.comppsych.2009.03.001 19932826

[B15] Ramacciotti CE Perrone P Burgalassi A Conversano C Massimetti G Dell’Osso L Orthorexia nervosa in the general population: a preliminary screening using a self-administered questionnaire (ORTO-15) Eat Weight Disord 2011 16 127 130 10.1007/BF03325318 21989097

[B16] Kinzl JF Hauer K Traweger C Kiefer I Orthorexia nervosa. Eine häufige Essstörung bei Diätassistentinnen? Ernahrungs-Umschau 2005 11 436 439

[B17] Kinzl JF Hauer K Traweger CH Kiefer I Orthorexia nervosa in dieticians Psychother Psychosom 2006 75 395 396 10.1159/000095447 17053342

[B18] Korinth A Schiess S Westenhoefer J Eating behaviour and eating disorders in students of nutrition sciences Public Health Nutr 2010 13 32 37 Az űrlap teteje 10.1017/S1368980009005709 19433007

[B19] Donini LM Marsili D Graziani MP Imbriale M Cannella C Orthorexia nervosa: a preliminary study with a proposal for diagnosis and an attempt to measure the dimension of the phenomenon Eat Weight Disord 2004 9 2 151 157 10.1007/BF03325060 15330084

[B20] Varga M Dukay-Szabó S Túry F van Furth EF Evidence and gaps in the literature on orthorexia nervosa Eat Weight Disord 2013 18 103 111 10.1007/s40519-013-0026-y 23760837

